# Lateralized effects of deep brain stimulation in Parkinson’s disease: evidence and controversies

**DOI:** 10.1038/s41531-021-00209-3

**Published:** 2021-07-22

**Authors:** Zhengyu Lin, Chencheng Zhang, Dianyou Li, Bomin Sun

**Affiliations:** 1 Department of Neurosurgery, Ruijin Hospital, Shanghai Jiao Tong University School of Medicine, Shanghai, China; 2 Center for Functional Neurosurgery, Ruijin Hospital, Shanghai Jiao Tong University School of Medicine, Shanghai, China; 3 Institute of Clinical Neuroscience, Ruijin Hospital LuWan Branch, Shanghai Jiao Tong University School of Medicine, Shanghai, China; 4 Shanghai Research Center for Brain Science and Brain-Inspired Intelligence, Shanghai, China

**Keywords:** Parkinson's disease, Therapeutics

## Abstract

The bilateral effects of deep brain stimulation (DBS) on motor and non-motor symptoms of Parkinson’s disease (PD) have been extensively studied and reviewed. However, the unilateral effects—in particular, the potential lateralized effects of left- versus right-sided DBS—have not been adequately recognized or studied. Here we summarized the current evidence and controversies in the literature regarding the lateralized effects of DBS on motor and non-motor outcomes in PD patients. Publications in English language before February 2021 were obtained from the PubMed database and included if they directly compared the effects of unilateral versus contralateral side DBS on motor or non-motor outcomes in PD. The current literature is overall of low-quality and is biased by various confounders. Researchers have investigated mainly PD patients receiving subthalamic nucleus (STN) DBS while the potential lateralized effects of globus pallidus interna (GPi) DBS have not been adequately studied. Evidence suggests potential lateralized effects of STN DBS on axial motor symptoms and deleterious effects of left-sided DBS on language-related functions, in particular, the verbal fluency, in PD. The lateralized DBS effects on appendicular motor symptoms as well as other neurocognitive and neuropsychiatric domains remain inconclusive. Future studies should control for varying methodological approaches as well as clinical and DBS management heterogeneities, including symptom laterality, stimulation parameters, location of active contacts, and lead trajectories. This would contribute to improved treatment strategies such as personalized target selection, surgical planning, and postoperative management that ultimately benefit patients.

## Introduction

Cerebral lateralization refers to the functional specialization of the two cerebral hemispheres^1^. For instance, the left cerebral cortex is dominant for motor control and verbal processing, whereas the right cerebral cortex is dominant for spatial cognition, body schema, proprioceptive control, and inhibition control^2^. The basal ganglia—a group of subcortical nuclei—is critical for information integration and processing of the cortex input for motor and cognitive functions^3^. Thus, it is postulated that the functions of basal ganglia are hemisphere-specific as well. Indeed, both neurobiological^4,5^ and structural^6^ basis as well as the electrophysiological pattern asymmetry^7^ suggest the functional laterality of basal ganglia. Therefore, interventions to left versus right basal ganglia may demonstrate distinctive effects on motor and cognitive features.

Parkinson’s disease (PD) is the second most common neurodegenerative disorder characterized by a selective and progressive loss of dopaminergic neurons in the substantia nigra, resulting in a dopamine deficiency in the basal ganglia^8^. Deep brain stimulation (DBS) is a well-established neurosurgical treatment for controlling motor symptoms and reducing levodopa-induced complications in advanced PD^9,10^. Currently, two main structures of the basal ganglia, the subthalamic nucleus (STN) and the globus pallidus interna (GPi), are primarily targeted in DBS surgery^11^. Bilateral and symmetric placement of DBS is the strategy adopted in the majority of cases as patients with advanced PD often show bilateral disabling motor symptoms^12^. Thus, bilateral effects of DBS on motor and non-motor symptoms have been extensively investigated^11,13^. In contrast, the unilateral effects—in particular, the potential lateralized effects of left- versus right-sided DBS, given the postulated functional lateralization of basal ganglia—have not been adequately recognized or studied. Moreover, to the best of our knowledge, no systematic review has been composed regarding this issue. In this review, therefore, we focus on the evidence and controversies regarding the potential lateralized effects of DBS on motor and non-motor symptoms in PD. We also highlight limitations of the current literature and potential factors that may influence the interpretation of the evidence.

## Results

### Motor features

Whether the unilateral left- and right-sided DBS have different effects on motor symptoms in patients with PD remains debated. Though the lateralization effect is not observed in all patients^14–16^, several studies have suggested that there exists a superiority of unilateral STN DBS in improving motor functions^17,18^. Schulz et al. enrolled 12 right-handed PD patients with stable bilateral STN DBS and reported that, in the off-medication condition, unilateral left-sided and bilateral STN stimulation resulted in better improvement in non-lateralized motor endpoints and overall motor scores than unilateral right-sided stimulation^17^. However, by studying 45 PD patients treated with bilateral STN DBS, Tabbal et al. found no differences between unilateral left- and right-sided STN stimulations in the improvement of gait speed in the off-medication state^14^. The differences between unilateral left- and right-sided stimulations on the improvement of rigidity and bradykinesia were not reported in this study, because authors focused mainly on discrepancies in the effects of ipsilateral and contralateral stimulation on these appendicular motor features^14^. Similarly, a comparable amelioration of the motor score and the working memory performance (see “neurocognition and speech” section) was described by Hershey et al. between unilateral left- and right-sided STN stimulation conditions in 49 PD patients with bilateral STN DBS in the off-medication state^15^. Instead, they suggested a potential causal role of the disease asymmetry to explain the different effects of unilateral STN DBS between the more and less affected side on motor functions and working memory performance^15^. Interestingly, although Lizarraga et al. reported no differences in motor and gait scores in the off-medication state between unilateral left- and right-sided stimulation conditions in 22 PD patients with the presence of the gait dysfunction after receiving bilateral STN DBS, they showed that unilateral right-sided STN stimulation might produce slightly greater improvement in gait kinematics (i.e., stride length) than left-sided stimulation^18^. They later postulated that the right (non-dominant) hemisphere may be dominant for axial motor control^2^. Castrioto et al. also reported no significant difference between unilateral left- and right-sided stimulation conditions on motor scores in 22 PD patients with bilateral STN DBS in the off-medication condition. However, they proposed the presence of a ‘dominant-STN’, the stimulation of which may lead to improvements of motor symptoms, similar to bilateral stimulation^16^. In this study, 11 (50%) participants presented a ‘dominant-STN’, in 8 of whom the dominant-STN was contralateral to the most affected side. Interestingly, the ‘dominant-STN’ phenomenon was associated with longer disease duration and tremor predominance^16^. A subsequent study further demonstrated that the unilateral stimulation of the ‘dominant-STN’ could offer improvement in gait parameters, which were comparable to those after bilateral stimulation. Whereas in patients without the ‘dominant-STN’, only bilateral stimulation could significantly improve gait parameters (*n* = 10)^19^. However, it is currently difficult to preoperatively select patients with underlying ‘dominant-STN’.

As mentioned above, Tabbal et al. reported that unilateral STN DBS reduced rigidity and bradykinesia both ipsilaterally and contralaterally^14^. However, the difference between unilateral left- and right-sided DBSs on ipsilateral motor effects was not investigated in this work. In fact, few studies in the current literature focused on this issue. Shemisa et al. investigated 73 right-handed PD patients treated with either unilateral GPi (*n* = 26) or STN (*n* = 47) DBS, and this study provided limited evidence of a similar ipsilateral motor improvement in the off-medication state after either unilateral left- or right-sided DBS stimulation, regardless of target^20^. This study, however, should be interpreted in caution as the target selection was not randomized and could be biased by several baseline factors.

### Neurocognition and speech

Lateralized deficits in cognitive performance, especially in language-mediated functions, have been recognized in the era of lesional surgeries for PD^21–23^. Compared with patients with right-sided lesions, PD patients who underwent left globus pallidotomy are more likely to experience postoperative declines in verbal fluency^21,23^. However, in the era of DBS surgeries, the lateralized effects of DBS on cognitive functions of PD have not been as adequately investigated as the effects of bilateral stimulation.

For GPi DBS, Vingerhoets et al. compared the cognitive outcomes at the 3-month follow-up to that at the preoperative baseline in a cohort of PD patients who received unilateral left- (*n* = 13) or right-sided (*n* = 7) GPi DBS in the on-medication condition^24^. Six patients who underwent the unilateral left-sided GPi DBS showed a tendency for cognitive decline (as measured by cognitive impairment index), which was not observed in patients who had received right-sided GPi DBS^24^. In another study, 6 PD patients were consecutively recruited to investigate the changes in cognitive performance after staged bilateral GPi DBS in the on-medication condition^25^. Among the 5 patients who received left-sided stimulation first, 4 showed a decline in semantic fluency. In contrast, only 1 of the 5 patients showed a further decline after the second, right-sided surgery^25^. Rothlind et al. included both staged bilateral GPi (*n* = 23) and STN (*n* = 19) DBS cases to evaluate cognitive outcomes in the on-medication condition. Participants were randomized to receive either staged bilateral GPi or STN DBS to avoid the selection bias. Authors reported that the deleterious effect of unilateral DBS on semantic fluency were largely due to the left-sided treatment^26^. Patients who initially received right-sided DBS displayed a significant decline in semantic fluency only after left-sided surgery^26^. Moreover, Zahodne et al. compared the cognitive performance of 22 PD patients with unilateral GPi (*n* = 12) or STN (*n* = 10) DBS to that of 19 PD controls^27^. They found that the unilateral DBS surgery was associated with declines in letter and semantic verbal fluency in the on-medication condition and suggested that such changes may be more common after left-sided DBS^27^. In contrast, Tröster et al. stated that in PD patients who received unilateral pallidal surgery, the decline in on-medication semantic verbal fluency was not associated with the side of surgery^28^. However, this finding should be interpreted prudently, as both pallidotomy (23 left-sided, 12 right-sided) and DBS (7 left-sided, 3 right-sided) surgeries were combined in the final analysis to increase the statistical power^28^.

Similarly, whether STN DBS has a potential lateralized effect on cognitive functions for PD remains controversial. In a preliminary study, Lueken et al. systematically evaluated the performance in the Wisconsin Card Sorting Test of 8 right-handed PD patients with bilateral STN DBS in the on-medication condition, and left- and right-sided stimulation was given in a counterbalanced manner. Compared to right-sided stimulation, selected measures of executive functions were more compromised under left-sided stimulation^29^. However, a subsequent study did not report any association between changes in the working memory capacity (i.e., spatial delayed response, SDR) and the side of STN stimulation in 49 PD patients with bilateral STN DBS in the off-medication condition^15^. Instead, the authors demonstrated that stimulation on the more affected side of the brain aggravated the SDR, whereas stimulation on the less affected side did not, suggesting that clinical asymmetry might interact with STN DBS to impact behavioral responses^15^. David et al. used a memory-guided sequential reaching task to examine the role of STN DBS in the intensive as well as integrative and coordinative aspects of motor control in 10 right-handed PD patients treated with bilateral STN DBS in the off-medication condition. They reported that unilateral right-sided STN stimulation significantly increased finger latency and reduced finger velocity (i.e., the intensive aspect) when compared to left-sided stimulation, but the endpoint error (i.e., the integrative and coordinative aspects) was similar between the two stimulation conditions^30^. One study that investigated cognitive-motor functions in prosaccade and antisaccade tasks in 10 right-handed PD patients with bilateral STN DBS in the off-medication state also found no difference in the spatiotemporal or the cognitive aspects of oculomotor control between unilateral left- and right-sided STN stimulations^31^.

The lateralized effects of STN DBS on changes in verbal fluency (dominant hemisphere) and visuospatial attention (non-dominant hemisphere) are also a subject of debate^17,26,27,32–35^. Concordant with the findings from Rothlind et al. and Zahodne et al. as mentioned above, Schulz et al. also suggested that the declines in syntactic and lexical language performance, speech rate, and laryngeal–articulatory coordination for PD patients with bilateral STN DBS may be principally due to the left-sided stimulation^17^. Sjöberg et al. compared the verbal fluency of 6 PD patients receiving unilateral left-sided STN DBS to that of 10 PD patients receiving bilateral STN DBS in the on-medication condition. At approximately 1.5-year follow-up, the bilateral STN DBS showed a more deleterious effect on both letter and category verbal fluency. A reasonable explanation is that the group receiving unilateral DBS may represent patients who were at an earlier stage of the disease, which may influence their neurocognitive performance^32^. More recently, Yilmaz et al. reported no significant discrepancy in verbal fluency or visual orientation between unilateral left- and right-sided stimulations in 29 right-handed PD patients with bilateral STN DBS in the on-medication condition^33^. In contrast, both Witt et al. (*n* = 12) and Schmalbach et al. (*n* = 13) reported a mild but significant shift of visuospatial attention towards the right side and a neglect of left-sided visual stimuli under unilateral stimulation of the left STN, similar to the neglect syndrome after right hemispheric lesions. Such declines in visuospatial attention could be corrected by stimulating the right STN^34,35^.

The difference in the inhibition control performance between unilateral left- and right-sided STN stimulations has also been investigated^36–38^. Ray et al. reported that the performance in stop-signal task declined after left-sided STN stimulation compared to that after right-sided treatment in 16 right-handed PD patients with bilateral STN DBS in the on-medication condition^36^. In contrast, Mirabella et al. did not find any difference in the performance in stop-signal task between unilateral left- and right-sided STN stimulations by evaluating 10 right-handed PD patients with bilateral STN DBS in the off-medication condition^37^. More recently, Mancini et al. recruited 20 right-handed PD patients with unilateral STN DBS (10 left-sided, 10 right-sided) and also reported no main effect of stimulating lateralization on the on-medication performance in countermanding task^38^. However, the discrepancies in findings between these studies should be carefully interpreted, because the PD cohort selection (i.e., participants with bilateral or unilateral DBS) and the medication status (i.e., off- or on-medication condition) during the task were different across studies, which might limit the interpolation of the results.

Regarding other speech-related functions, Santens et al. reported negative effects of unilateral left-sided STN DBS on prosody and articulation (and hence intelligibility) by separately stimulating the left and right STN in 7 PD patients with bilateral STN DBS in the off-medication state^39^. Similar results have also been reported by other groups^17,40,41^. Wang et al. examined the speaking rate and articulatory accuracy of syllable repetitions in 20 right-handed PD patients with unilateral STN DBS (10 left-sided, 10 right-sided) before the surgery and at 3- to 6-month follow-up after withdrawal of anti-parkinsonian medication. They concluded that a decrease in articulatory accuracy and speaking rate was associated with unilateral left-sided STN DBS^40^. They also reported a significant decline in vocal intensity and vowel duration baseline in 3 right-handed PD patients with unilateral left-sided STN DBS at the 3-month follow-up. Such a decline was not observed in patients with unilateral right-sided STN DBS^41^.

### Neuropsychiatry

To date, few studies have assessed the lateralized effects of DBS on mood in PD patients, and findings were not conclusive. Campbell et al. reported that left-sided STN DBS may produce greater mood improvement than right-sided STN DBS^42^. They measured the mood response with a computerized version of the Visual Analog Scale in 24 PD patients with bilateral STN DBS. Left-sided stimulation resulted in significantly greater amelioration in valence than right-sided stimulation, and trend-level significance was seen for apathy ratings. No significant differences were observed for anxiety ratings or emotional arousal between unilateral left- and right-sided stimulations^42^. Birchall et al. reported a similar improvement in depressive symptoms at the 6-month follow-up compared to the preoperative baseline between PD patients with unilateral left-sided (*n* = 26) STN stimulation and those with unilateral right-sided (*n* = 24) STN stimulation^43^. In another study, the authors prospectively assessed the apathy score change preoperatively and postoperatively at the 6-month follow-up in PD patients with either unilateral GPi (11 left-sided, 4 right-sided) or unilateral STN (20 left-sided, 13 right-sided) DBS, and concluded that the incidence of postoperative apathy was not associated with the laterality of GPi or STN DBS^44^.

### Sleep

Interestingly, Amara et al. measured the short-term effect of unilateral STN DBS on sleep quality in 53 PD patients (28 left-sided, 25 right-sided)^45^. They reported that the improvement in subjective sleep quality at the 6-month follow-up was greater in right-sided stimulation group than in the left-sided group. However, the preoperative baseline sleep quality score was also worse in the right-sided stimulation group. Therefore, it is possible that the reported results were due to more easily detectable improvements in patients with worse baseline sleep dysfunction rather than a direct lateralized stimulating effect of the treatment^45^. To the best of our knowledge, no other studies have been published to support or oppose this finding.

## Potential confounding factors

### Study design

To compare the differential effects of unilateral left- versus right-sided DBS in PD patients, several studies^20,24,27,28,38,40,43–45^ have analyzed two separate cohorts with respective unilateral left- and right-sided DBS implantation. One study also compared the group with unilateral left-sided STN DBS with that with bilateral treatment^32^. Consequently, differences in baseline characteristics between the two groups may limit the interpretation of the results. Other studies^14–19,25,26,29–31,33–37,42^ recruited PD patients with simultaneous or staged bilateral DBS and collected the outcome data under unilateral on-stimulation conditions in a randomized or counterbalanced manner. Among these, some studies applied randomized stimulation in consecutive days to ensure a sufficient ‘washout’ interval. However, some required patients to complete tests under different stimulating conditions on the same day with a shorter ‘washout’ period. In addition, the medication conditions (i.e., on- or off-medication) also differed across studies. For example, the majority of the studies that evaluated the potential lateralized stimulating effects on motor outcomes were conducted in the off-medication condition to exclude the interference of drug effects. In contrast, several studies investigating the cognitive outcomes reported their results in the on-medication state to reduce the possibility for motor symptoms to limit the neurocognitive performance. Actually, current literature suggests both beneficial and detrimental cognitive effects of levodopa in PD^46^. Moreover, if the analysis included a comparison between the pre- and post-operative status in the on-medication condition, the different management of postoperative medication could be another confounding factor. Last but not least, the follow-up periods in different studies ranged from months to years, and several studies only assessed the clinical outcomes in a subacute setting. Therefore, the discrepancies in the study design may considerably limit the interpretation and comparison of the literature.

### Baseline characteristics

One of the prominent clinical characteristics in PD is an asymmetrical distribution of motor symptoms at the onset and during the disease course^47^. The asymmetrical loss of dopaminergic innervation in the striatum has been suggested as the root of the symptom laterality in PD. Because of the hemispherical lateralization of several cognitive and affective functions and the potential role of impaired dopaminergic transmission in non-motor symptoms in PD, several studies have been focused on the correlation between the symptom laterality and the prevalence and severity of several aspects of cognitive and neuropsychiatric domains in PD^48–50^. For instance, one literature review concluded that right-sided motor symptom predominance (i.e., a putative left-sided hemisphere dominant dopamine deficiency) was associated with deficits in language-related functions and verbal memory in PD patients. In contrast, patients with left-sided motor symptom predominance (i.e., a putative right-sided hemisphere dominant dopamine deficiency) showed worse performance in tasks of spatial attention, visuospatial orienting, and memory and mental imagery^48^. However, several studies, failed to identify such a relationship in patients with early-stage unmedicated PD^49,50^. These studies suggest a negative effect of dopamine-replacement therapy on the development of lateralized cognitive deficits in relation to the symptom laterality. Nevertheless, symptom laterality serves as a non-negligible factor for the interpretation of the putative lateralization of DBS efficacy in PD.

Furthermore, right- or left-handedness—a critical phenotype for hemispheric language dominance^51–53^—was not systematically and objectively analyzed in the articles included in this review. We suggest that the precise and individualized determination of cerebral dominance would be beneficial for delineating the potential lateralized effects of DBS in PD.

### Trajectory and location of lead contacts

The surgical trajectory and electrode placement in relation to the STN topography are critical sources of variation in postoperative outcome in PD^54–58^. For example, York et al. reported that declines of several aspects of neuropsychological outcomes after STN DBS in PD patients may be related to the trajectory as well as electrode location in a hemisphere-specific manner^54^. Witt et al. also indicated an association between surgical trajectory through the head of the caudate nuclei and declines in global cognitive performance in PD patients with STN DBS^55^. Specifically, Tripoliti et al. found that the medially located electrodes in the left STN and the high stimulating intensity of the left electrode were significantly associated with the poor outcome of the speech intelligibility after bilateral STN DBS surgery^57^. More recently, Petry-Schmelzer et al. reported that the inter-individual variability of non-motor outcomes (e.g., mood/apathy, attention/memory, and sleep outcomes) after STN DBS in PD patients may depend on the location of neurostimulation^58^.

Moreover, the number of microelectrode recording (MER) trajectories has been hypothesized to contribute to cognitive declines following DBS surgery, because the degree of local injury along the trajectory or at the target site would increase as the number of MER passes increase^59^. Limited evidence suggests that the microlesional effect could contribute to the early transient verbal fluency decline following DBS surgery, while the long-term fluency impairment might be related to the direct effect of STN DBS^60^. Later, two retrospective studies concordantly reported that the postoperative cognitive change at approximately 1-year follow-up was not correlated with the number of MER trajectories^59,61^. Prospective studies are warranted to confirm these findings in the future.

### Stimulation parameters

Varying stimulating parameters (i.e., contact, stimulating intensity, frequency, and pulse width) may also considerably affect the treatment efficacy and introduce adverse effects in PD^62,63^. As mentioned above, Tripoliti et al. presented a correlation between the high stimulating intensity of the left electrode and the result of poor speech intelligibility after bilateral STN DBS surgery^57^. Moreover, severe gait disturbances and freezing episodes often appear in patients with long-term high-frequency DBS^64^. Evidence suggested an amelioration of gait disorders with low-frequency (60 Hz) STN stimulation in PD^64,65^. In addition, several studies showed that a lateralized STN neuromodulation (i.e., unilateral reduction of stimulating intensity) can also affect the gait disturbance and posture in PD^66,67^. For example, Fasano et al. showed that reducing the STN stimulation voltage on the contralateral side of the leg with a longer step length could improve the frequency and duration of gait freezing^66^. Twenty-two participants with bilateral STN DBS were assessed in four stimulation conditions (i.e., off-stimulation, bilateral on-stimulation, unilateral on-stimulations) in a subacute setting, while the chronic effect of stimulating intensity reduction on axial and appendicular motor functions was not investigated. More recently, a PD case with Pisa syndrome following chronic bilateral STN DBS successfully treated with left-sided stimulating intensity reduction was reported by Lizarraga et al.^67^. The lateral trunk flexion angle reduced from 12° to 4° by reducing the left-STN stimulation voltage from 4.8 to 3.8 V^67^. Although these findings are preliminary and should be interpreted with caution given the methodological drawbacks and limited sample size, the potential feasibility of the lateralized STN neuromodulation strategy suggests the presence of a functional lateralization of the basal ganglia.

## Limitations

The main limitation of this comprehensive review relies on the overall low-quality of the current literature investigating the potential lateralized effects of DBS in PD. The majority of the studies were conducted on small samples, possibly due in part to the substantial burden of clinical evaluation under various stimulus conditions. Most of the studies assessed clinical outcomes only in a subacute setting or at a short-term follow-up. Few studies used or specified a randomized and blinded evaluation paradigm to minimize biases. Furthermore, potential confounding factors discussed above were not well addressed in the current literature, which considerably limited the interpolation of the findings. For instance, effects of location of active contacts and trajectories on neurocognitive outcomes were poorly discussed in the literature involved in this review. Furthermore, authors paid little attention to the correlation between changes in motor and non-motor symptoms when investigating the presumed lateralized DBS effects. This could be interesting because a lack of correlation might suggest different underlying mechanisms of the potential lateralized DBS effects on motor and non-motor domains of PD.

## Conclusions and future directions

There is insufficient evidence in current literature to draw solid conclusions about the lateralized effects of DBS on motor and non-motor outcomes in PD. Several factors, such as the inherent bias in experimental design, symptom laterality, and location of active contacts with respect to the putative topography of the nuclei, surgical trajectory, and DBS programming strategies should be considered for the accurate interpretation of results. Nevertheless, limited evidence suggests potential lateralized effects of STN DBS on both axial motor symptoms and language-related functions in PD.

To date, most of the studies have focused on STN DBS, and the potential efficacy of lateralization of GPi DBS in PD has not been adequately investigated. In addition, whether STN and GPi DBS exert differential lateralization effects on motor and non-motor outcomes in PD also warrants further investigation. Ongoing research focusing on delineating the lateralized effects of DBS can lead to a better understanding of the therapeutic mechanisms of DBS in PD. This would potentially contribute to improving treatment strategies such as personalized target selection (e.g., bilateral asymmetric implant^68^), surgical planning, and postoperative management that ultimately benefits patients.

## Methods

Ethical approval and patient consent were not required for this study. The main inclusion criteria were articles that directly compared the efficacy of unilateral left- to right-sided DBS on motor or non-motor symptoms in PD. Publications in English language before February 2021 were initially searched in PubMed database by using the following search criteria: ((((((((left[Title/Abstract]) OR (right[Title/Abstract])) OR (sided[Title/Abstract])) OR (left-sided[Title/Abstract])) OR (right-sided[Title/Abstract])) OR (laterality[Title/Abstract])) AND (deep brain stimulation[MeSH Terms])) AND (parkinson’s disease[MeSH Terms])) AND (english[Language]). The search results were verified manually. Of the 243 publications, 225 articles were excluded after title and abstract screening. Subsequently, the full texts of potentially relevant publications were analyzed. The references, citations, and similar articles suggested in PubMed for the included articles were also screened for additional eligible studies. The literature search was conducted by Z. Lin and C. Zhang. Finally, after duplicate removal, 27 publications were included in this review (Table 1). The quality of the publications was classified according to the Classification of Evidence Schemes of the Clinical Practice Guideline Process Manual of the American Academy of Neurology^69^.

**Table 1:** Overall study characteristics and outcomes.

Study	Level of evidence^a^	Patients	Handedness (R/L)	Study design	Interpretation L vs. R
Schulz et al. (2012)^17^	III	12 BL STN	12/0	Blinded, randomized STIM-OFF vs. R vs. L vs. BL under MED-OFF, at least 24 h for washout	Greater motor score improvement but worsened speech and language performance in L condition
Tabbal et al. (2008)^14^	III	52 BL STN	Not specified	Double-blinded, randomized STIM-OFF vs. R vs. L vs. BL under MED-OFF, at least 30–42 min for washout	Similar gait speed improvement, quantitative differences in rigidity and bradykinesia not reported
Hershey et al. (2008)^15^	III	49 BL STN	46/3	Double-blinded, randomized STIM-OFF vs. R vs. L vs. BL under MED-OFF, at least 60 min for washout	Similar motor improvement and working memory performance
Lizarraga et al. (2016)^18^	II	22 BL STN	20/2	Double-blinded, randomized STIM-OFF vs. R vs. L vs. BL under MED-OFF, at least 60 min for washout	Similar motor score improvement but slightly smaller improvement in gait kinematics (stride length) in L condition
Castrioto et al. (2011)^16^	II	22 BL STN	21/1	Double-blinded, randomized STIM-OFF vs. R vs. L vs. BL under MED-OFF, at least 60 min for washout	Similar motor improvement
Rizzone et al. (2017)^19^	II	10 BL STN	Not specified	BL vs. blinded, randomized R vs. L vs. STIM-OFF under MED-OFF, at least 60 min for washout	Similar motor improvement in patients without presumed ‘dominant-STN’
Shemisa et al. (2011)^20^	III	16 L GPi 10 R GPi 29 L STN 18 R STN	73/0	4-month Postop vs. Preop under MED-OFF	Similar ipsilateral motor improvement regardless of target
Vingerhoets et al. (1999)^24^	III	13 L GPi 7 R GPi	Not specified	3-month Postop vs. 2-month Preop under MED-ON	Tendency of cognitive decline in L group
Fields et al. (1999)^25^	IV	6 Staged BL GPi	5/1	3-month 2nd Postop vs. 2-month 1st Postop vs. 1-month Preop under MED-ON	Tendency of decline in verbal fluency with L treatment
Rothlind et al. (2007)^26^	III	23 Staged BL GPi 19 Staged BL STN	20/3 (GPi) 16/3 (STN)	15-month 2nd Postop vs. 6-month 1st Postop vs. Preop under MED-ON	Significant decline in verbal fluency with L treatment
Zahodne et al. (2009)^27^	III	7 L GPi 5 R GPi 7 L STN 3 R STN	Not specified	Postop vs. Preop under MED-ON	Significant decline in verbal fluency in L group regardless of target
Tröster et al. (2002)^28^	III	30 L GPi^b^ 15 R GPi^b^	40/5	4-month Postop vs. 1-momth Preop under MED-ON	Similar decline in verbal fluency
Lueken et al. (2008)^29^	III	8 BL STN	8/0	Counterbalanced R vs. L, average interval of 16 days between both sessions	Decline in selected aspects of executive functions in L condition
David et al. (2018)^30^	III	10 BL STN	10/0	Counterbalanced STIM-OFF vs. R vs. L vs. BL under MED-OFF in 4 consecutive days, 3 h for washout	Similar performance in integrative and coordinative aspects of motor control
Goelz et al. (2017)^31^	III	10 BL STN (10 HC)	10/0 (10/0)	Counterbalanced STIM-OFF vs. R vs. L vs. BL under MED-OFF in 4 consecutive days, 3 h for washout	Similar spatiotemporal and cognitive aspects of oculomotor control
Sjöberg et al. (2012)^32^	III	6 L STN 10 BL STN	16/0	1.5-year Postop vs. 6-month Postop vs. Preop under MED-ON	Significantly less decline in verbal fluency in L group than in BL group
Yilmaz et al. (2015)^33^	III	29 BL STN	29/0	Blinded, randomized STIM-OFF vs. R vs. L vs. BL under MED-ON, 3 min for washout	Similar performance in verbal fluency
Witt et al. (2006)^34^	II	12 BL STN	12/0	Blinded, randomized STIM-OFF vs. R vs. L vs. BL under MED-OFF, at least 30 min for washout	Increased reaction time of both hand movement to visual stimuli in the left extrapersonal hemispace in L condition, reversible in BL condition
Schmalbach et al. (2014)^35^	II	13 BL STN	Not specified	Double-blinded, randomized R vs. L vs. BL under MED-ON, at least 30 min for washout	Decreased oculomotor exploration time of the extrapersonal space in L condition, reversible in BL condition
Ray et al. (2009)^36^	III	16 BL STN (10 HC)	16/0	Counterbalanced STIM-OFF vs. R vs. L under MED-ON, 10–15 min for washout	Significantly worse response inhibition performance in L condition
Mirabella et al. (2012)^37^	II	10 BL STN (13 HC)	10/0	Counterbalanced STIM-OFF vs. R vs. L vs. BL under MED-OFF on different days, 60 min for washout	Similar performance in the stop-signal task
Mancini et al. (2018)^38^	II	10 L STN 10 R STN (22 HC)	20/0 (22/0)	Counterbalanced Postop STIM-OFF vs. STIM-ON under MED ON, 60 min for washout	Similar performance in the stop-signal task
Santens et al. (2003)^39^	III	7 BL STN	Not specified	Blinded, randomized STIM-OFF vs. R vs. L vs. BL under MED-OFF, 10 min for washout	Significantly worse performance of prosody, articulation, and hence intelligibility, in L condition
Wang et al. (2006)^40^	III	10 L STN 10 R STN	20/0	Double-blinded, randomized 3- to 6-month Postop STIM-OFF vs. Postop STIM-ON vs. 1-month Preop under MED-OFF, at least 30 min for washout	More decline in articulatory accuracy and speaking rate in L group
Wang et al. (2003)^41^	IV	3 L STN 3 R STN	6/0	Double-blinded 3-month Postop STIM-OFF vs. Postop STIM-ON vs. Preop under MED-OFF	Significant decline in vocal intensity and vowel duration in L group
Campbell et al. (2012)^42^	II	24 BL STN	Not specified	Double-blinded, counterbalanced STIM-OFF vs. R vs. L vs. BL under MED-OFF, at least 42 min for washout	Significantly greater improvement in valence, trend-level greater improvement in apathy in L condition
Birchall et al. (2017)^43^	III	26 L STN 24 R STN	Not specified	Postop (3-, 6-month) vs. Preop	Similar improvements in depressive symptoms
Kirsch-Darrow et al. (2011)^44^	III	11 L GPi 4 R GPi 20 L STN 13 R STN (48 PD control)	Not specified	Postop (2-, 4-, 6-month) vs. Preop	Similar incidence of postoperative apathy regardless of target
Amara et al. (2012)^45^	III	28 L STN 25 R STN	Not specified	Postop (3-, 6-month) vs. Preop	Significantly more improvement in subjective sleep quality in R group

^a^According to the Classification of Evidence Schemes of the Clinical Practice Guideline Process Manual of the American Academy of Neurology.

^b^Consists of both cases undergoing pallidotomy or pallidal deep brain stimulation.

Abbreviations: *BL* bilateral stimulation, *L* left-sided stimulation, *R* right-sided stimulation, *STIM-OFF* off-stimulation, *STIM-ON* on-stimulation, *STN* subthalamic nucleus, *GPi* globus pallidus interna, *HC* healthy control, *Postop* postoperative, *Preop* preoperative, *MED-ON* on-medication, *MED-OFF* off-medication.

## Data Availability

No datasets were generated or analyzed during the current study.
